# Additive growth inhibitory effects of ibandronate and antiestrogens in estrogen receptor-positive breast cancer cell lines

**DOI:** 10.1186/bcr1363

**Published:** 2005-12-12

**Authors:** Fabrice Journe, Carole Chaboteaux, Nicolas Magne, Hugues Duvillier, Guy Laurent, Jean-Jacques Body

**Affiliations:** 1 Laboratory of Endocrinology and Bone Diseases and Department of Internal Medicine, Institut J Bordet, Centre des Tumeurs de l'Université Libre de Bruxelles, Brussels, Belgium; 2 Department of Radiotherapy, Institut J Bordet, Centre des Tumeurs de l'Université Libre de Bruxelles, Brussels, Belgium; 3 Laboratory of Experimental Hematology, Institut J Bordet, Centre des Tumeurs de l'Université Libre de Bruxelles, Brussels, Belgium; 4 Laboratory of Histology, Faculty of Medicine and Pharmacy, Université de Mons-Hainaut, Mons, Belgium

## Abstract

**Introduction:**

Bisphosphonates are inhibitors of osteoclast-mediated tumor-stimulated osteolysis, and they have become standard therapy for the management of bone metastases from breast cancer. These drugs can also directly induce growth inhibition and apoptosis of osteotropic cancer cells, including estrogen receptor-positive (ER+) breast cancer cells.

**Methods:**

We examined the anti-proliferative properties of ibandronate on two ER+ breast cancer cell lines (MCF-7 and IBEP-2), and on one ER negative (ER-) cell line (MDA-MB-231). Experiments were performed in steroid-free medium to assess ER regulation and the effect of ibandronate in combination with estrogen or antiestrogens.

**Results:**

Ibandronate inhibited cancer cell growth in a dose- and time-dependent manner (approximate IC_50_: 10^-4 ^M for MCF-7 and IBEP-2 cells; 3 × 10^-4 ^M for MDA-MB-231 cells), partly through apoptosis induction. It completely abolished the mitogenic effect induced by 17β-estradiol in ER+ breast cancer cells, but affected neither ER regulation nor estrogen-induced progesterone receptor expression, as documented in MCF-7 cells. Moreover, ibandronate enhanced the growth inhibitory action of partial (4-hydroxytamoxifen) and pure (ICI 182,780, now called fluvestrant or Faslodex™) antiestrogens in estrogen-sensitive breast cancer cells. Combination analysis identified additive interactions between ibandronate and ER antagonists.

**Conclusion:**

These data constitute the first *in vitro *evidence for additive effects between ibandronate and antiestrogens, supporting their combined use for the treatment of bone metastases from breast cancer.

## Introduction

Over 80% of women suffering from advanced breast cancer ultimately develop bone metastases [1,2]. As revealed by observations published more than a decade ago [3], patients with estrogen receptor (ER)-positive neoplasms are more prone to develop skeletal secondaries. Metastatic breast cancer cells stimulate osteoclast-mediated bone resorption, inducing a marked osteolysis that is responsible for considerable morbidity [4,5].

Bisphosphonates are potent inhibitors of osteoclast-mediated osteolysis [6] and have, therefore, emerged as a rational approach for the management of bone metastases [7,8]. These drugs are synthetic analogs of pyrophosphate. They show high affinity for bone mineral and preferentially accumulate at sites of active bone remodeling. The most potent bisphosphonates are nitrogen-containing compounds (e.g. ibandronate, zoledronic acid) that interfere with the mevalonate pathway, leading to inhibition of the post-translational prenylation of proteins [9,10]. From cell culture studies, it is known that they inhibit the resorptive activity and induce the apoptosis of mature osteoclasts [10,11].

Moreover, there is now compelling *in vitro *evidence that bisphosphonates may also act directly on tumor cells. They inhibit proliferation and induce apoptosis in cell lines derived from various neoplasms, such as breast [12,13] and prostate carcinomas [14,15]. Bisphosphonates may also antagonize the growth stimulation induced by bone-derived growth factors on human breast cancer cells [16]. Furthermore, recent animal data indicate that bisphosphonates inhibit bone metastasis growth through promotion of apoptosis in cancer cells [17,18]. Bisphosphonates also reduce tumor cell invasiveness [19] and cell adhesion to bone [20].

In the clinical setting, bisphosphonates are often combined with conventional endocrine agents for the treatment of patients with metastatic bone disease, especially as endocrine therapy is often preferred to chemotherapy for patients with soft tissue and bone metastases [21]. The extent to which such bisphosphonate and antiestrogen combination affects tumor cell growth has not yet been examined, however, and it is unknown which interactions are operating. The triphenylethylene antiestrogen tamoxifen is the classic hormonal treatment for the management of breast cancers expressing ERs [22]. On the other hand, ICI 182,780 [23] (now called fulvestrant or Faslodex™) is the only steroidal antiestrogen that has reached clinical development [24]. Both compounds are competitive inhibitors for the binding of 17β-estradiol (E_2_) to ER, but their mechanisms of action are quite different [25]. Tamoxifen, a partial ER antagonist, inhibits the activation function-2 (AF-2)-mediated transactivation, probably via the recruitment of corepressors [26,27]. Yet this type of antagonist does not interfere with AF-1-mediated transactivation. Tamoxifen, as well as its active metabolite 4-hydroxytamoxifen, has also been shown to cause ER nuclear accumulation [28]. By contrast, ICI 182,780, a pure ER antagonist, suppresses both AF-1 and AF-2 ER transactivation functions, and prevents nuclear transport of the receptor [29]. In addition, such pure antagonists reduce the half-life of ER protein, leading to a decrease in receptor content (down-regulation) [30].

In the present study, we assessed the anti-proliferative properties of ibandronate, a newly developed nitrogen-containing bisphosphonate, on ER-positive breast cancer cells. These *in vitro *experiments were conducted in steroid-free medium (SFM) to allow for the assessment of estrogenic responses and for the measurement of ER content and activity. Besides, it is known that ER antagonists exert a growth-inhibitory effect on MCF-7 cells even in the absence of estrogenic stimulation [31-34]. We thus tested ibandronate in combination with antiestrogens in order to identify possible additive or synergistic interactions.

## Materials and methods

### Cell culture conditions

The ER-positive MCF-7 breast cancer cell line (ATCC HTB-22) was initially obtained in 1977 from the Michigan Cancer Foundation (Detroit, MI, USA). The IBEP-2 cell line was previously established in our laboratory from a pleural effusion due to metastatic breast carcinoma [35] and also expresses functional ER. MDA-MB-231 breast carcinoma cells (ATCC HTB-26) lack ER expression.

All experiments were performed in plastic flasks, dishes and multi-well plates obtained from Nunc (Naperville, IL, USA). Cells were cultured at 37°C in a humidified 95% air and 5% CO_2 _atmosphere. For routine maintenance, cells were cultured in 75 cm^2 ^flasks containing RPMI medium 1640 (Gibco BRL, Life Technologies, Merelbeke, Belgium) with Phenol Red, supplemented with 10% (v/v) heat-inactivated FCS, and containing standard concentrations of L-glutamine, penicillin and streptomycin (Gibco BRL). Cells were harverested by trypsinization (0.05% (w/v) trypsin, 0.53 mM EDTA.4Na) twice a week. For experiments, cells were plated in SFM made up of RPMI medium 1640 without Phenol Red supplemented with 10% (v/v) FCS stripped of endogenous estrogens by a dextran-coated charcoal treatment as previously described [36]. One day later, the seeding medium was replaced by fresh SFM containing ibandronate (gift from Hoffmann-LaRoche (Basel, Switzerland), E_2 _(Sigma, St Louis, MO, USA), 4-hydroxytamoxifen (Sigma), ICI 182,780 (Tocris, Bristol, UK) or vehicle for 1 to 6 days.

### Crystal violet staining

Cell number was assessed indirectly by staining with crystal violet dye as previously described [37]. Briefly, cancer cells were seeded in 96-well plates (density 5,000 cells/well) in SFM, and cultured for 24 h. Cells were then exposed to compounds or vehicle at various concentrations and time incubations as described in Results. Medium was removed, cells were gently washed with PBS, fixed with 1% (v/v) glutaraldehyde/PBS for 15 minutes and stained with 0.1% crystal violet (w/v in ddH_2_O) for 30 minutes. Cells were destained under running tap water for 15 minutes and subsequently lysed with 0.2% Triton X-100 (v/v in ddH_2_O). The absorbance was measured at 550 nm using a Microplate Autoreader EL309 (BIO-TEK Instruments, Winooski, VT, USA). Blank wells lacked cells and drugs. The IC_50 _value refers to drug concentrations producing 50% inhibition of growth.

### Cell count

Cell growth was also assessed by cell count. Cells were plated in 12-well dishes at a density of 10^4 ^cells/cm^2 ^in SFM. At day 1, the seeding medium was replaced by fresh SFM containing 10^-4 ^M ibandronate and/or 10^-8 ^M E_2_. After three days of incubation, cells were dislodged from the vessel bottom by treatment with a trypsin-EDTA solution. After vigorous pipetting, concentrations of cells in suspension were determined in an electronic cell counter (model Z1 Coulter counter, Beckman Coulter, Fullerton, CA, USA).

### Apoptosis determination

Apoptotic cell death was assessed using annexin-V fluorescein isothiocyanate (FITC) and propidium iodide double staining (Apo*Target*™, Annexin-V FITC Apoptosis Kit, BioSource Europe, Nivelles, Belgium), according to the manufacturer's recommendations. This method is based on apoptosis-related cell membrane modifications and relies on selective binding of annexin-V to phosphatidylserine expressed in the outer membrane leaflet during the early stages of apoptosis. Propidium iodide staining reveals cell surface membrane permeability associated with necrosis or late stage of apoptosis. Briefly, MCF-7 cells were seeded in 6-well plates (density 50,000 cells/well) in SFM, and cultured for 24 h. Cells were then exposed to compounds or vehicle for 3 to 6 days at concentrations as described in Results. Medium was renewed at day 3. Cells were washed twice in PBS, harvested by treatment with a trypsin-EDTA solution, centrifuged and resuspended in 100 μl annexin-V binding buffer. Cell suspensions received 5 μl of FITC-labeled annexin-V and 10 μl of propidium iodide buffer, were incubated for 15 minutes at room temperature in darkness, and, finally, were diluted with 400 μl annexin-V binding buffer. Cells were then analyzed by using a flow cytometrer (FACSCalibur, Becton Dickinson, Franklin Lakes, NJ, USA). Data are presented as dot plots showing fluorescence intensity of annexin-V FITC versus propidium iodide. Percentages of apoptotic cells are percentages of annexin-V positive and propidium iodide negative cells.

### Western blot analysis

ER and progesterone receptor (PgR) amounts were determined by western blotting. Cells were plated in 60 cm^2 ^petri dishes (density 10,000 cells/cm^2^) in SFM, cultured for 24 h and then incubated with compounds or vehicle as specified in Results. Cell monolayers were harvested and lysed using detergent cocktail, as previously described [37]. Solubilized proteins were subjected to western blotting using polyclonal rabbit anti-human ERα antibody (HC-20, Santa Cruz Biotechnology, Santa Cruz, CA, USA) diluted 1:5,000, or monoclonal mouse anti-human PgR (A/B isoforms) antibody (NCL-PGR-AB, Novocastra Laboratories, Newcastle upon Tyne, UK) diluted 1:500. Peroxidase-labeled donkey anti-rabbit IgG antibody (1:5,000) or peroxidase-labeled sheep anti-mouse IgG antibody (1:5,000) (Amersham Pharmacia Biotech, Roosendaal, The Netherlands) were used depending on the primary antibody. The bound peroxidase activity was revealed using the Lumi-Light Western Blotting Substrate (Roche, Mannheim, Germany). The immunoreactive band intensity was estimated using a computer-assisted gel scanning densitometer (GS-710 Callibrated Imaging Densitometer) and Quantity One software, both from Bio-Rad (Hercules, CA, USA).

### Protein determination

Protein concentrations of total cell lysates were determined by the BCA Protein Assay (Pierce, Rockford, IL, USA) using bovine serum albumin as standard.

### Combination index calculations

The cytotoxic effects obtained with the different combinations of ibandronate and 4-hydroxytamoxifen or ICI 182,780 were evaluated according to the method of Chou and Talalay [38] on Calcusyn software (Biosoft, Cambridge, UK). This method allows the identification of interactions between two drugs, regardless of the mechanism of action of the individual drugs. Cells were incubated with increasing concentrations of ibandronate (10^-6 ^to 10^-3 ^M) alone and in combination with increasing concentrations of 4-hydroxytamoxifen (10^-10 ^to 10^-7 ^M) or ICI 182,780 (10^-10 ^to 10^-7 ^M) for 72 h. Interaction between the double combinations was assessed by means of an automatically computed combination index (CI). CI was determined at 50% and 75% cell growth, and was defined as follows:

CI_A+B _= [(D_A/A+B_)/D_A_] + [(D_B/A+B_)/D_B_] + [α(D_A/A+AB _× D_B/A+B_)/D_A_D_B_]

where CI_A+B _= CI for a fixed effect (F) for the combination of cytotoxic A and cytotoxic B; D_A/A+B _= concentration of cytotoxic A in the combination A + B giving an effect F; D_B/A+B _= concentration of cytotoxic B in the combination A + B giving an effect F; D_A _= concentration of cytotoxic A alone giving an effect F; D_B _= concentration of cytotoxic B alone giving an effect F; α = parameter with value 0 when A and B are mutually exclusive and 1 when A and B are mutually non-exclusive.

The CI indicates synergism for values lower than 0.8, additivity for values included between 0.8 and 1.2, and antagonism for values higher than 1.2. Values of 0.8 and 1.2 suggest slight synergistic and additive cytotoxic activities, respectively.

### Statistical analysis

Data are reported as means ± standard deviation (SD). Statistical analysis was performed by analysis of variance (ANOVA) which assumes a similar SD in different groups. Thus, a logarithmic transformation was applied before ANOVA when SD values were found to differ statistically. Dunnett *post hoc *test was used to compare treated conditions to the untreated condition (control) and Turkey *post hoc *test was performed for multiple comparisons between groups. The level of statistical significance was arbitrarily set at 0.01. All analyses used SPSS software (Paris, France).

## Results

### Effects of ibandronate on breast cancer cell growth in steroid-free medium

**Figure 1:**
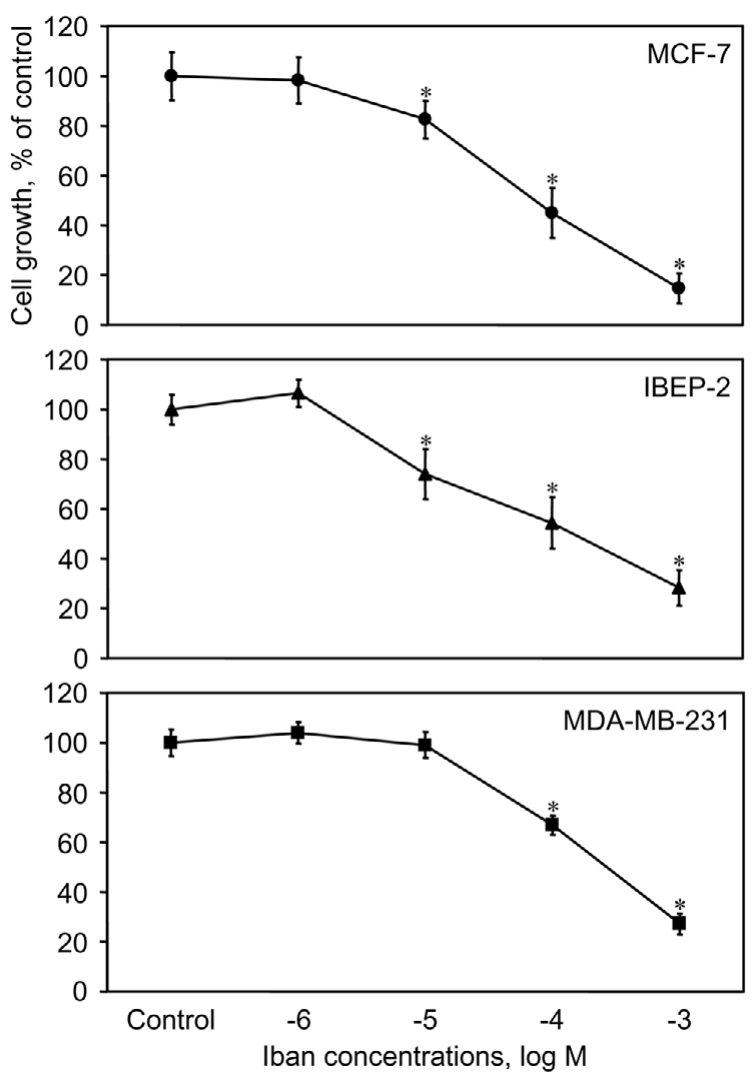
Dose-response analyses of ibandronate on MCF-7, IBEP-2 and MDA-MB-231 cells. Breast cancer cells were cultured for three days in steroid-free medium containing ibandronate (Iban) or vehicle (control). Cell proliferation was determined by crystal violet staining assay. Data are presented as percentages of control values (mean ± SD). Experiments were performed four times in replicate. Asterisks indicate ANOVA p < 0.01 versus control, Dunnett *post hoc *test.

MCF-7 cells were plated in RPMI 1640 medium supplemented with charcoal-stripped fetal bovine serum (SFM), and cultured for 24 h before exposure for 72 h to ibandronate at concentrations ranging from 10^-6 ^to 10^-3 ^M. In these conditions, ibandronate inhibited cell growth in a concentration-dependent manner, as assessed by photometry after crystal violet staining (approximate IC_50 _10^-4 ^M) (Fig. 1, upper panel). Electronic cell count after trypsinization gave quite similar results on hormone-deprived MCF-7 cells when the effect of 10^-4 ^M ibandronate was evaluated after 3 days of treatment (Table 1). IBEP-2 cells exhibited a similar dose-response curve to ibandronate as assessed by crystal violet staining (approximate IC_50 _10^-4 ^M), while MDA-MD-231 cells were slightly less sensitive to the bisphosphonate (approximate IC_50 _3 × 10^-4 ^M) (Fig. 1, middle and lower panels).

**Figure 2:**
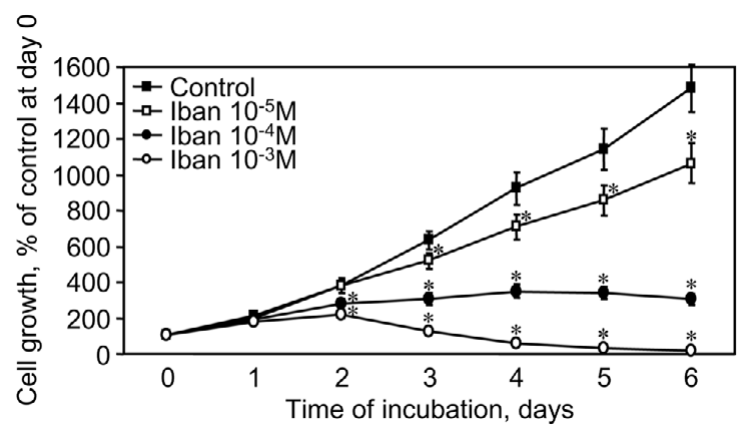
Time-course experiments of ibandronate (Iban) on MCF-7 cells. Cells were cultured for up to six days in steroid-free medium containing Iban or vehicle (control). Cell growth was determined as described in Fig. 1. Statistical analysis on log transformed data; asterisks indicate ANOVA p < 0.01 versus control at the same day, Dunnett *post hoc *test.

As shown by time-course experiments over 6 days, ibandronate at 10^-5 ^M only produced a weak inhibition of MCF-7 cell growth, detectable from day 4. By contrast, higher drug concentrations drastically affected cell growth kinetics: 10^-4 ^M ibandronate induced rapid cytostatic effects up to day 6, whereas 10^-3 ^M ibandronate clearly exerted cytotoxic effects (Fig. 2).

**Figure 3:**
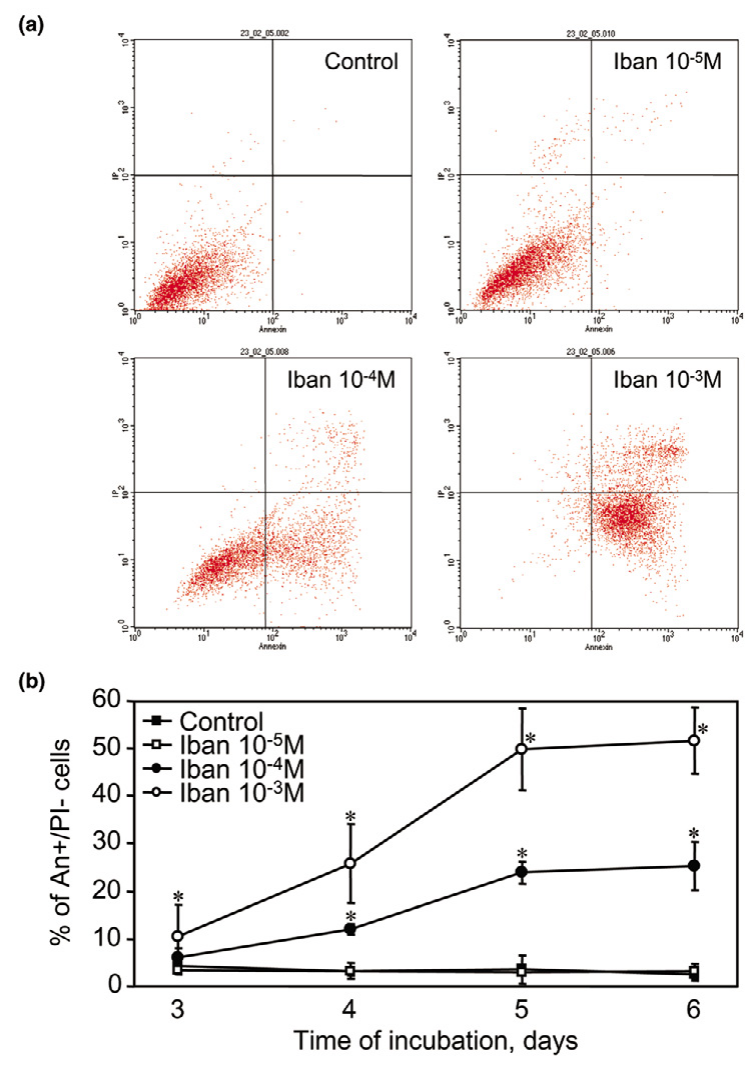
Ibandronate-induced apoptosis in MCF-7 cells assessed by annexin-V fluorescein isothiocyanate and propidium iodide double staining (An/PI). Cancer cells were cultured for three to six days in steroid-free medium containing ibandronate (Iban) or vehicle (control). Apoptotic cells were detected as An+/PI- cells, while viable cells were An-/PI- cells and necrotic cells were PI+. **(a) **Representative dot plots after five days of Iban exposure. **(b) **The percentage of An+/PI- cells was determined from three experiments (mean ± SD). Statistical analysis on log transformed data; asterisks indicate ANOVA p < 0.01 versus corresponding control, Dunnett *post hoc *test.

Moreover, high ibandronate concentrations induced apoptotic cell death, as documented by the detection of annexin-positive and propidium iodide-negative MCF-7 cells (Fig. 3a). The percentages of annexin-positive cells after 5 days of incubation with 10^-4 ^M and 10^-3 ^M ibandronate were 23.5 ± 2.4 (mean ± SD) and 50.0 ± 8.7, respectively, indicating that the inhibition of cell growth was caused, at least in part, by cell apoptosis (Fig. 3b). Of note, 10^-4 ^M ibandronate did not induce significant apoptosis at day 3. No significant cell death was observed using 10^-5 ^M bisphosphonate.

**Figure 4:**
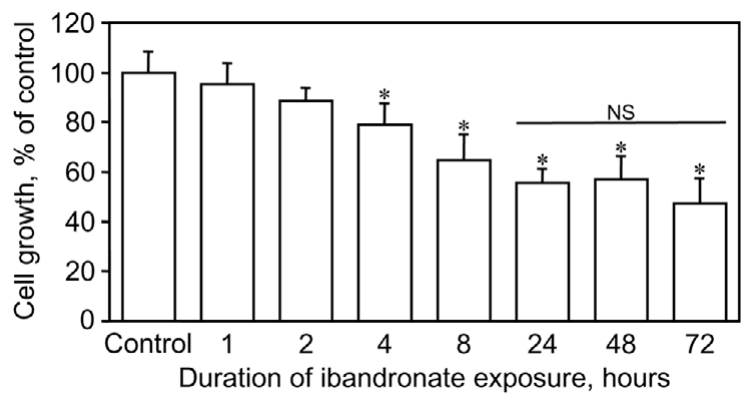
Effects of various durations of exposure of MCF-7 cells to ibandronate. Cells were cultured in steroid-free medium and exposed to 10^-4 ^M ibandronate or vehicle (control) for 1 to 72 h (pulse exposures). Cell growth measurements were performed after 72 h as described in Fig. 1. Asterisks indicate ANOVA p < 0.01 versus control, Tukey *post hoc *test. NS, not significant.

The influence of increasing treatment duration on cell growth estimated at 72 h is illustrated by pulse exposure studies in Fig. 4. A 2 h exposure to ibandronate did not significantly affect cell proliferation, whereas bisphosphonate exposure for 4 h resulted in a significant decrease in cell growth by 21%. In addition, a 45% inhibition of cell growth was already seen after a 24 h treatment and the effect was not significantly greater when the incubation with ibandronate was prolonged up to 48 or 72 h (43% and 53% growth inhibition, respectively). This suggests that the growth inhibition recorded after a 24 h exposure is already irreversible and that 24 to 72 h exposure does not augment inhibitory efficacy.

### Influence of ibandronate on the mitogenic effect of E_2_, and on estrogen receptor regulation and activity in MCF-7 cells

**Figure 5:**
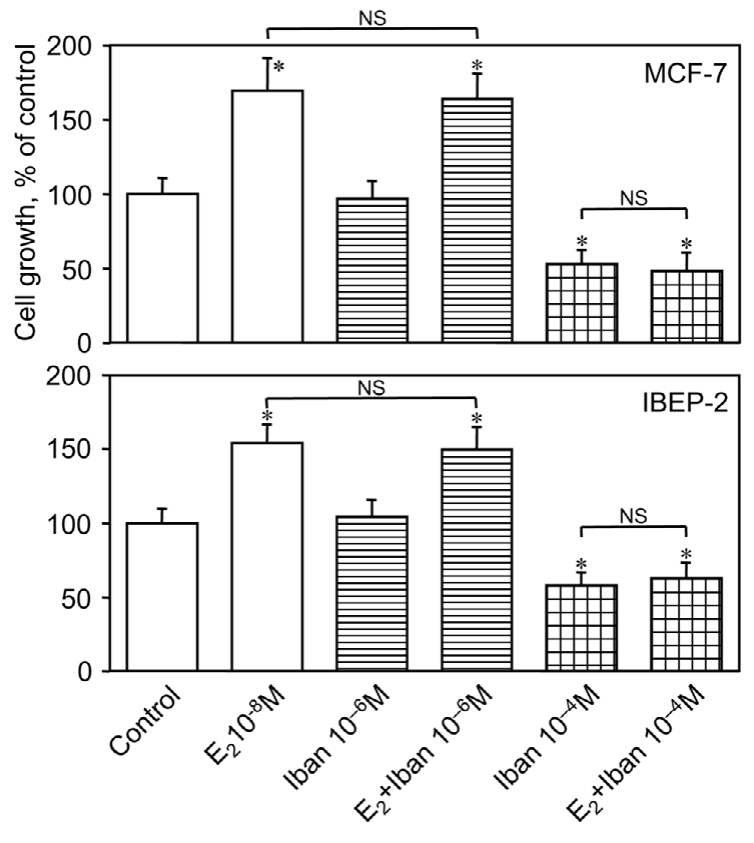
Effects of ibandronate (Iban) on the stimulation of MCF-7 and IBEP-2 cell growth induced by 17β-estradiol (E_2_). Cells were incubated with 10^-4 ^M Iban and/or 10^-8 ^M E_2 _or vehicle (control) for 3 days. Cell growth determination was performed after three days as described in Fig. 1. Asterisks indicate ANOVA p < 0.01 versus control, Tukey *post hoc *test. NS, not significant.

As previously reported [39], 10^-8 ^M E_2 _stimulated the proliferation of MCF-7 cells in SFM (Fig. 5, upper panel; Table 1). In this context, 10^-6 ^M ibandronate, which failed to change basal cell proliferation, did not affect the growth stimulation induced by E_2_. By contrast, 10^-4 ^M ibandronate completely abolished the mitogenic effect induced by estrogenic stimulation, and the growth inhibitory effects of ibandronate were unaffected by the presence of E_2_. Closely similar findings were obtained using IBEP-2 cells, which have been recently reported as estrogen-responsive cells resembling MCF-7 cells [39] (Fig. 5, lower panel), indicating that the inhibition by ibandronate of the mitogenic stimulation induced by E_2 _was not restricted to MCF-7 cells.

**Figure 6:**
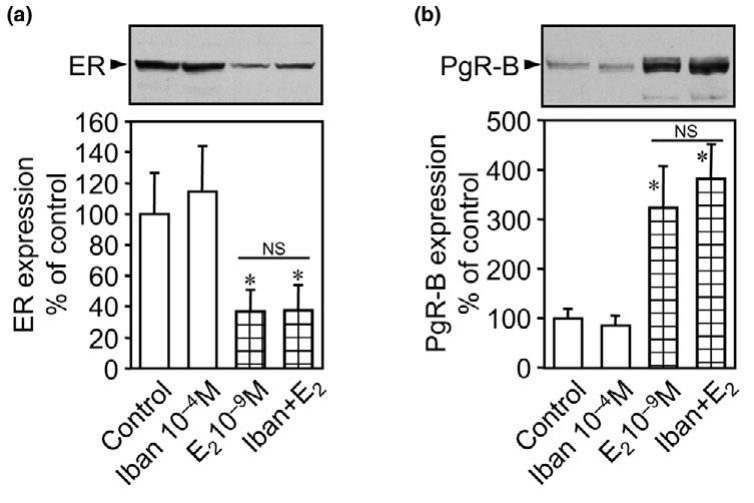
Estrogen receptor (ER) and progesterone receptor (PgR) expression in MCF-7 cells exposed to ibandronate (Iban) and/or 17β-estradiol (E_2_) as assessed by western blot. **(a) **For ER determination, MCF-7 cells were incubated for 24 hours with 10^-4 ^M Iban and/or 10^-9 ^M E_2 _or vehicle (control) in steroid-free medium. Equal quantities of proteins (20 μg) were subjected to SDS-PAGE and electrotransferred onto nitrocellulose membranes. Immunodetection was performed with anti-human ER antibody raised against its F domain. **(b) **For PgR measurement, cells were incubated for 72 hours in the same experimental conditions. Equal amounts of proteins (40 μg) were loaded onto SDS-PAGE and immunoblot was revealed using anti-human PgR antibody raised against A/B isoforms of the receptor. Blots show representative experiments performed at least twice. Data were obtained from densitometric analyses and are presented as mean percentages of control values. Asterisks indicate ANOVA p < 0.01 versus control, Tukey *post hoc *test. NS, not significant.

ER expression and activity were examined in the same culture conditions by western blot analysis. Ibandronate alone had no effect on ER content whereas E_2 _induced a marked receptor down-regulation reflected by a 63% decline in ER steady-state level after 24 h (Fig. 6a). Ibandronate did not affect ER decrease induced by E_2_. The expression of PgR was also investigated as it is estrogen-inducible and viewed as a classic marker of ER activation [40]. Thus, the effect of ibandronate on PgR expression was assessed by western blot analysis using an antibody raised against the A/B isoforms of PgR. Exposure of MCF-7 cells for 72 h to 10^-9 ^M E_2 _increased by more than three-fold the level of PgR B isoform (Fig. 6b). Ibandronate modified neither PgR baseline level nor the increase in PgR level induced by E_2_. As previously reported [39], the A isoform of PgR was not detectable in untreated MCF-7 cells and only a small amount of this isoform was observed after E_2 _stimulation.

Overall, these data indicate that ibandronate completely blocks the proliferative response induced by E_2_, without affecting ER regulation and activity. From these results, it can be inferred that ibandronate does not directly act on the ER pathway but interferes with E_2_-induced mitogenicity at steps downstream or independent of ER-mediated signaling. Indeed, in MCF-7 cells exposed to ibandronate, ER kept its transactivation ability.

### Combination of ibandronate and antiestrogens

**Figure 7:**
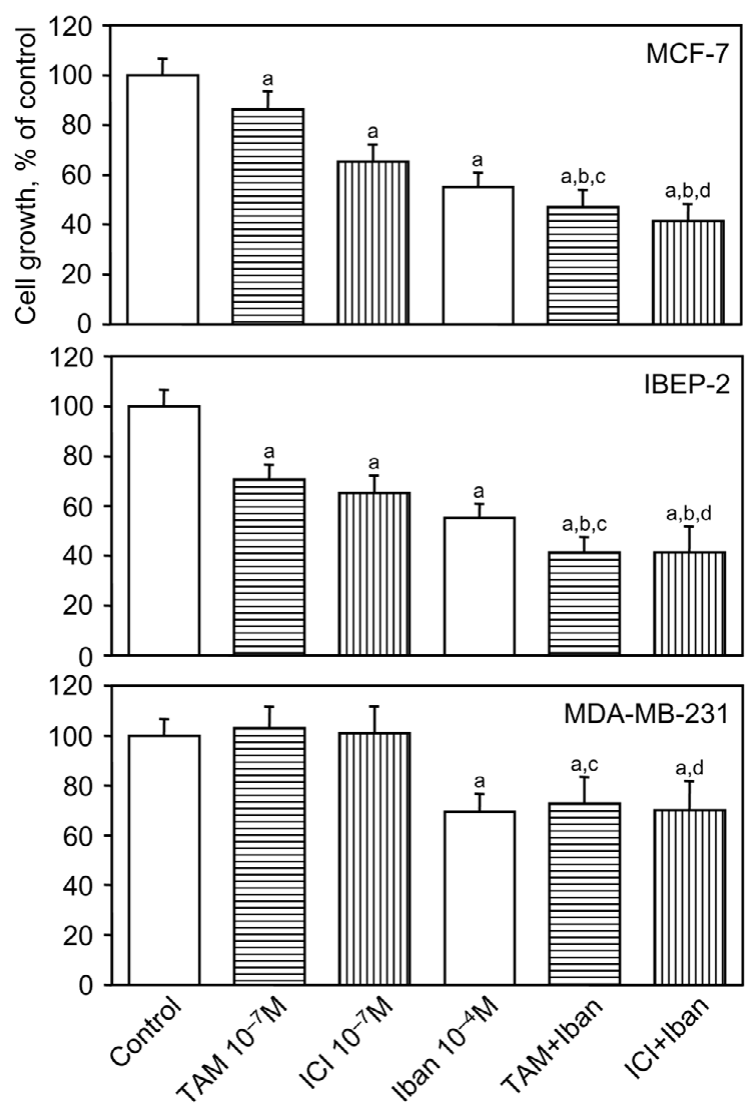
Effect of combined treatment of MCF-7, IBEP-2 and MDA-MB-231 cells with ibandronate (Iban) and 4-hydroxytamoxifen (TAM) or ICI 182,780 (ICI). Cells were incubated with 10^-4 ^M Iban in the presence or absence of 10^-7 ^M TAM or ICI for 72 hours. Cell proliferation was determined as described in Fig. 1. ANOVA, p < 0.01: a, significantly different from control; b, significantly different from Iban alone; c, significantly different from TAM alone; d, significantly different from ICI alone; Tukey *post hoc *test.

We assessed the effects of ibandronate in combination with antiestrogens. Data shown above indeed suggest that bisphosphonate-treated MCF-7 cells keep a functional form of ER, which should be able to promote the anti-proliferative action of ER antagonists. Two well-known antiestrogens were used for this study: an active metabolite of the partial antiestrogen 4-hydroxytamoxifen and the pure antiestrogen ICI 182,780. In preliminary studies, these drugs alone or in combination with ibandronate were tested at fixed concentrations with regard to their inhibitory effects on MCF-7 growth in steroid-free conditions. Antiestrogens alone (10^-7 ^M) significantly decreased cell growth by 14% and 35% for 4-hydroxytamoxifen and ICI 182,780, respectively (Fig. 7, upper panels). Importantly, the growth inhibitory action induced by both antiestrogens was significantly enhanced by ibandronate. Thus, when using combined treatments, 4-hydroxytamoxifen plus ibandronate decreased cell growth by 51%, and ICI 182,780 plus ibandronate inhibited cell proliferation by 58%. Similarly, the growth of the ER-positive IBEP-2 cells was also inhibited by both antiestrogens, and growth inhibition was again significantly higher when ER antagonists were combined with ibandronate (Fig. 7, middle panel). By contrast, antiestrogens did not affect the proliferation of the ER-negative MDA-MB-231 cells, and they did not change the growth inhibitory effect of ibandronate (Fig. 7, lower panel). These data indicate that antiestrogen response in sensitive breast cancer cells may be enhanced by ibandronate.

**Figure 8:**
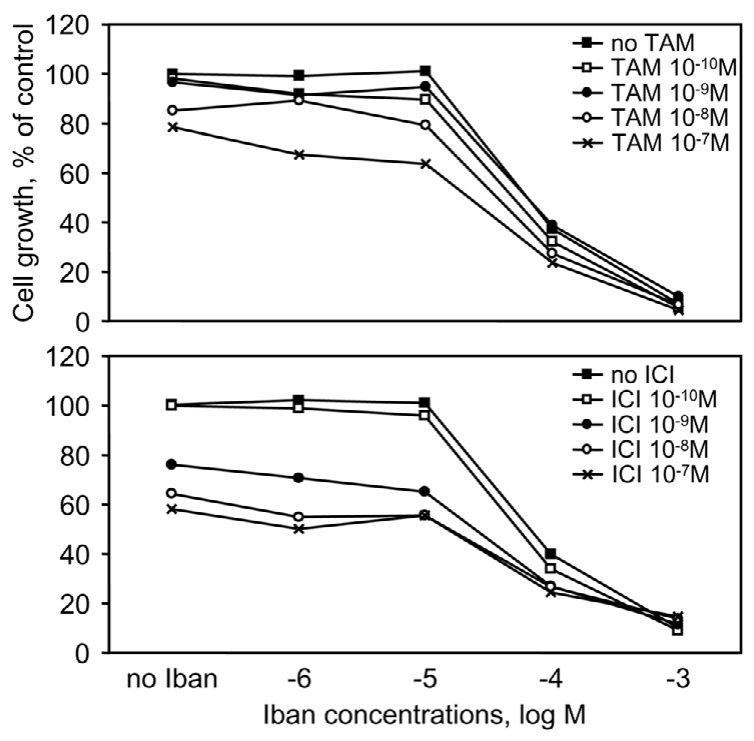
Dose-response curves of ibandronate (Iban from 10^-6 ^to 10^-3 ^M) in combination with increasing concentrations of 4-hydroxytamoxifen (TAM from 10^-10 ^to 10^-7 ^M) or ICI 182,780 (ICI from 10^-10 ^to 10^-7 ^M). MCF-7 cells were incubated for 72 hours. Cell proliferation was determined by crystal violet staining assay. Data are presented as percentages of control values (mean) and were used for combination index calculations (see Table 2). Experiments were performed three times in replicate.

To better characterize the interactions between ibandronate and ER antagonists in MCF-7 cells, drug combinations were evaluated over a wide range of concentrations (from 10^-6 ^to 10^-3 ^M for ibandronate and from 10^-10 ^to 10^-7 ^M for the antiestrogens). Dose-response curves are illustrated in Fig. 8. These data were submitted to isobolographic analysis to calculate the CIs at 50% and 75% inhibition of cell growth, according to the analytical procedure developed by Chou and Talalay [38]. Results are summarized in Table 2. According to the CI values, the effects of ibandronate and antiestrogen were additive, suggesting that these compounds act through distinct mechanisms.

## Discussion

Bisphosphonates, including ibandronate, are widely used for the treatment of bone diseases involving enhanced osteoclast-mediated bone resorption, such as osteoporosis, tumor-induced hypercalcemia and, most importantly, cancer-induced bone disease [7,41]. Bisphosphonates thus constitute a major advance in the supportive care of cancer patients who develop skeletal metastases [42]. They exert a clinically significant analgesic activity and reduce by up to 40% the frequency of cancer-induced bone complications in patients with bone metastasis from breast cancer [43]. The beneficial effects of ibandronate, a new and potent bisphosphonate, in patients sufffering from breast cancer-induced osteolysis have recently been reported [44-46]. A substantial morbidity nevertheless persists even with the use of such new potent agents, indicating that bisphosphonates are not fully effective in blocking tumor-induced osteolysis. More studies are needed, notably to better understand the activity of bisphosphonates on tumor cells.

Recent data from *in vitro *work on breast cancer-derived cell lines indicate that nitrogen-containing bisphosphonates can directly inhibit tumor cell growth, primarily by inducing apoptosis [12,13,47-49]. However, these studies were performed in medium supplemented with whole serum, where the presence of steroids does not allow the evaluation of possible interactions of bisphosphonates with ER-mediated signaling. In the present study, we investigated the effects of ibandronate on the growth of MCF-7, IBEP-2 and MDA-MB-231 cells in medium supplemented with charcoal-stripped serum, providing a steroid-free environment suitable for the assessment of estrogenic responses. In fact, these experimental conditions may reflect more accurately the clinical situation because the vast majority of patients suffering from breast cancer are postmenopausal women with low circulating estrogen levels.

In the first part of this work, we showed that ibandronate (a nitrogen-containing bisphosphonate) induced a dose-dependent decrease in the growth of the three tested breast cancer cells cultured in SFM. These results were consistent with previous reports using steroid-containing medium (complete medium) [12,13,47], but contrast with our recent data showing that clodronate (a non-nitrogen-containing bisphosphonate) can stimulate the proliferation of MCF-7 cells in SFM [37]. In fact, this mitogenic effect of clodronate appears to be mediated by the activation of ERs and is completely suppressed by antiestrogens. Moreover, focusing on MCF-7 cells, we showed that 10^-3 ^M ibandronate exerted strong cytotoxicity partly through apoptosis induction. A lower concentration (10^-4 ^M) of ibandronate exerted cytostatic effects associated with moderate apoptosis induction, suggesting that cell proliferation was exactly balanced by cell death. Of note, significant apoptosis was only observed after four days of incubation with 10^-4 ^M ibandronate. Hence, at day 3, cell growth inhibition could be explained by cell cycle arrest, this step preceding apoptosis induction. Furthermore, in our experimental conditions, short-term exposures (four hours) of cells to 10^-4 ^M ibandronate were sufficient to induce a significant inhibition of MCF-7 cell proliferation. In addition, the presence of ibandronate for only 24 hours led to an irreversible loss of cell proliferative capacity, as shown by measurement two days later, and growth inhibition was comparable to a full-time exposure. This is consistent with the observations of Jagdev *et al*. [47] showing that incubation of MCF-7 cells with zoledronic acid, even for a short period of time, results in a significant reduction in cell number and a sizeable increase in apoptosis. These results might be important insofar as serum ibandronate concentrations are maintained for only a few hours after oral bisphosphonate administration [50] and raise the exciting possibility of short-term effects of bisphosphonates in non-osseous sites. On the other hand, in the particular microenvironment of bone metastases, bisphosphonates accumulate at the surface of bone resorption sites. This creates a compartment where cells undergo prolonged exposure to high drug concentrations. *In vivo *data revealed that effective local concentrations of bisphosphonates at sites of active bone resorption are much higher than serum levels, and may reach up to 10^-3 ^M in the resorption lacunae [51].

In the second part of our study, we examined the effects of ibandronate on estrogenic stimulation of MCF-7 and IBEP-2 cells. Interestingly, our experiments conducted in SFM showed that, at a concentration affecting cell proliferation (10^-4 ^M) but not at a lower concentration (10^-6 ^M), ibandronate suppressed the mitogenic effect induced by E_2 _and inhibited cell growth regardless of the presence of estrogen. As the nitrogen-containing bisphosphonate ibandronate is known to act through the inhibition of the mevalonate pathway and subsequent protein prenylation [9], the estrogenic stimulation of cell proliferation might require some prenylated proteins. Indeed, it has recently been reported that prenylated proteins play a role in estradiol-induced stimulation of cell proliferation through activation of the Src/Ras/Erk pathway [52]. Nevertheless, as shown by the current observations using MCF-7 cells, ibandronate had no direct effect on ER expression and did not affect E_2_-induced receptor down-regulation. Similarly, ibandronate did not affect the baseline level of progesterone receptor and did not interfere with E_2_-induced expression of this receptor. Altogether, these data indicate that ibandronate did not alter the regulation and the activity of ERs in MCF-7 cells, while it could totally prevent estrogen-induced cell proliferation in ER-positive breast cancer cells, suggesting that it acts downstream of ER-mediated gene transactivation.

Cancer therapy mostly relies on the use of drug combinations, which generally improve the therapeutic index, that is, give better responses with less toxicity. In this setting, bisphosphonates are most often used concomitantly with endocrine therapy. The presence of functional ERs in MCF-7 cells exposed to ibandronate suggests that these cells should remain sensitive to antiestrogenic agents. In the third part of our work, we thus tested the effects of ibandronate, antiestrogens (4-hydroxytamoxifen and ICI 182,780) and combinations thereof on MCF-7, IBEP-2 and MDA-MB-231 cell growth. Of note, our data show that antiestrogens alone inhibited the proliferation of MCF-7 and IBEP-2 cells in SFM. Used as a negative control, MDA-MB-231 cell growth was not affected by antiestrogens. These results confirm previous studies showing that ER antagonists are able to reduce the growth of ER-positive breast cancer cells, even in the absence of estrogenic stimulation [53]. It is conceivable that ER in estrogen-deprived MCF-7 cells is activated by phosphorylation due to cross-talk with other signaling pathways [54,55]. Thus, antiestrogens would completely abolish this activation. Moreover, when ER-positive breast cancer cells were exposed to ibandronate and antiestrogens, we observed additive inhibition of cell proliferation. The mechanism by which this occurred has not been fully elucidated but inhibition of the mevalonate pathway and subsequent protein prenylation due to ibandronate treatment, and prevention of cell proliferation by antiestrogens might result in this additive effect. Indeed, nitrogen-containing bisphosphonates are known to act as analogs of isoprenoid diphosphate lipids and to inhibit farnesyl pyrophosphate synthase, an enzyme of the mevalonate pathway [56,57]. This inhibition results in decreased isoprenoid lipid production (farnesyl pyrophosphate and geranylgeranyl pyrophosphate) and prevents protein prenylation [58]. On the other hand, antiestrogens are reported to cause the arrest of MCF-7 cells in the G1 phase of the cell cycle, resulting in a lower proportion of cells in S phase [59,60]. Moreover, cells treated with partial and pure antiestrogens show a significant decrease in cyclin D1 mRNA, which suggests that cyclin may be a target of antiestrogens, thus blocking entry into S phase [61]. Because of these different mechanisms of action, it is not a surprise that we found additive growth inhibitory effects when ibandronate and antiestrogens were added concomitantly. The mechanisms involved in the additive effect of ibandronate and antiestrogens are still elusive, however, and require further investigations.

Interestingly, in accordance with our results, recent data indicate that tamoxifen and the farnesyl transferase inhibitor FTI-277, acting through distinct pathways, exert an additive effect on MCF-7 cells, inhibiting cell cycle progression and cell proliferation [62]. Of note, farnesyl transferase functions closely downstream of farnesyl pyrophosphate synthase (the target for nitrogen-containing bisphosphonates) as it catalyzes the covalent attachment of farnesyl groups to prenylated proteins.

## Conclusion

Our results indicate that ibandronate inhibits breast cancer cell growth, both in the presence and absence of estrogenic stimulation. Moreover, the growth inhibition induced by classic antiestrogens, such as 4-hydroxytamoxifen and ICI 182,780, is larger on cancer cells expressing ERs when they are combined with ibandronate. These data suggest for the first time the existence of additive interactions between bisphosphonates and antiestrogens. Thus, our *in vitro *data provide a rationale for the combined use of ibandronate and antiestrogens in breast cancer patients suffering from bone metastases.

## Abbreviations

AF = activation function; CI = combination index; E_2 _= 17β-estradiol; ER = estrogen receptor; FCS = fetal calf serum; FITC = fluorescein isothiocyanate; PBS = phosphate-buffered saline; PgR = progesterone receptor; SD = standard deviation; SFM = steroid-free medium.

## Competing interests

The authors declare that they have no competing interests.

## Authors' contributions

FJ designed the experiments, performed the analysis and interpretation of the data and drafted the manuscript. CC carried out cell culture experiments, cell growth determination and western blot analysis. NM participated in combination index calculation. HD carried out apoptosis determination. GL carried out cell growth experiments and critically revised the manuscript. JJB participated in the design of the experiments, discussed the results and revised the manuscript. All authors read and approved the final manuscript

## Figures and Tables

**Table 1:** Cell growth determination

Experimental conditions^a^	Mean^b^	Standard deviation
Control	100.0	10.4
Iban 10^-4 ^M	59.3^c^	13.1
E_2 _10^-8 ^M	316.8^c^	65.4
E_2 _+ Iban	44.4^c,d^	14.7

Cell growth was determined by cell count, as detailed in Materials and methods. ^a^Exposure to ibandronate (Iban) and/or 17β-estradiol (E_2_) for 72 hours. ^b^Mean percentages of control values (mean ± SD) from three separate experiments. ^c^Statistical analysis (ANOVA, p < 0.01 versus control) on log transformed data, Tukey *post hoc *test. ^d^No significant difference compared with Iban and significantly different from E_2_.

**Table 2:** Correspondence of combination index values to combined drug effects in the MCF-7 cell line

Drug association^a^	Iban + TAM	Iban + ICI
50% CI, mean (SD)	1.0 (0.1)	0.9 (0.2)
Combination effect	Additive	Additive
75% CI, mean (SD)	1.1 (0.1)	1.0 (0.3)
Combination effect	Additive	Additive

The 50% and 75% combination index values corresponding to drug concentrations leading to 50% and 75% cell growth, respectively, were determined by crystal violet staining assay, as detailed in Materials and methods. ^a^Exposure to ibandronate (Iban) and/or 4-hydroxytamoxifen (TAM) or ICI 182,780 (ICI) for 72 hours in three separate experiments. SD, standard deviation.
